# Accessibility of pediatric inpatient services in Japan

**DOI:** 10.1371/journal.pone.0201443

**Published:** 2018-08-03

**Authors:** Akira Ehara

**Affiliations:** Faculty of Health Services Management, Hiroshima International University, Hiroshima, Hiroshima Prefecture, Japan; Universite de Bretagne Occidentale, FRANCE

## Abstract

In Japan, all citizens are covered by the national insurance system. Children’s medical expenses are subsidized by local government co-payments. This removed most economic barriers to visiting medical facilities, geographical obstacles to pediatric medical services remain, including distance to medical facilities and transportation time. However, information on geographic accessibility of pediatric inpatient services is scarce. In this study, I calculated the proportion of children resident in areas accessible to pediatric inpatient service providers within 30 and 60 minutes by automobile. Calculations were based on addresses of hospitals that met criteria for high reimbursement for secondary and tertiary pediatric inpatient services, data for residential blocks, and data for the average velocity of an automobile. In total, 88.0% of children lived within 30 minutes of these hospitals and 95.2% of children lived within 60 minutes. The percentage of children with such access was higher in regions with high population density (e.g., Kanto and Kinki) compared with regions with low population density (e.g., Hokkaido, Tohoku, and Shikoku). Furthermore, regions with high population density also had high rates of children that lived within reach of hospitals with at least five full-time pediatricians.

## Introduction

In Japan, all citizens are covered by the national insurance system [1]. Local governments subsidize co-payment of medical expenses for children [2]. Although these initiatives removed most economic barriers to visiting medical facilities, geographical obstacles to pediatric medical services remain, including distance to medical institutions and transportation time. Japanese citizens have free access to medical facilities. This system allows patients to choose any clinic or hospital. At night and during holidays, however, most patients are not able to visit their preferred clinics or hospitals because these facilities do not always provide medical services. Therefore, the geographic accessibility of the nearest hospitals must also be analyzed. To provide effective pediatric services, the Japanese government promoted concentration of medical resources into “regional pediatrics centers,” which provide pediatric inpatient services and primary care at night and during holidays [3]. The number of hospitals with pediatric departments decreased from 4,120 in 1990 to 2,656 in 2014 [4]. However, the accessibility of these facilities has not been comprehensively analyzed. Previously, I calculated the linear distance from each residential block in which children lived to the nearest hospital that met high reimbursement criteria for secondary and tertiary pediatric inpatient services, to show an index of accessibility [5]. Determining the time and distance involved in visiting medical facilities is necessary to discuss accessibility of such facilities. The Japanese government has reported average automobile velocities according to the type and width of roads in urban and rural areas [6]. This makes it possible to calculate the average time required to visit the closest pediatric inpatient service provider using geographic information systems.

In some hospitals, pediatric departments only provide pediatric outpatient services; it is unknown how many hospitals provide inpatient services for children. However, hospitals can receive high reimbursement for secondary and tertiary pediatric inpatient services if they meet health authority criteria for the allocation of full-time pediatricians and nurses [7]. There are five high reimbursement criteria for secondary pediatric inpatient services. Hospitals with at least one full-time pediatrician are able to receive medical fees based on one of these criteria. The criteria met by hospitals with pediatric departments provide an indicator of medical resources for pediatric inpatient care, including the number of pediatricians and nurses. Japan has no clear distinction between medical facilities for secondary and tertiary inpatient care; however, hospitals with many pediatricians are likely to provide tertiary medical services. Lists of these hospitals are available on regional health authority web pages.

Although the address of each child in Japan is unknown, the Population Census reports the number of children in 217,186 residential blocks [8]. When children need pediatric inpatient services, most guardians transport children to hospital by automobile, including private cars, taxis, and ambulances. In this study, I calculated the proportion of children resident in areas accessible to pediatric inpatient service providers within 30 and 60 minutes, using the address of hospitals that met the high reimbursement criteria, data for residential blocks, and data for the average velocity of an automobile.

## Materials and methods

The special hospital fee for secondary and tertiary pediatric inpatient services (Syouni-Nyuin-Iryou-Kanriryou in Japanese) is higher than the ordinary hospital fee, and is grouped in five criteria according to the allocation of full-time pediatricians and nurses (Table 1). This study used addresses for 803 hospitals that met these criteria, as listed on regional health authority web pages (at January 17, 2017, S1 Table).

**Table 1:** High reimbursement criteria for secondary and tertiary pediatric inpatient services.

Requirements	Criteria				
	1	2	3	4	5
Full-time pediatricians	≥20	≥9	≥5	≥3	≥1
Patients/nurse	≤7	≤7	≤7	≤10	≤15
Ward	Children only			Beds for children ≥10	-
Mean hospital stay (days)	<21	<22	<23	<28	-
Number of hospitals as at January 17, 2017	66	187	93	329	128

Japan is divided into 217,186 residential blocks under the Act on Indication of Residential Address. The mean area of these blocks is 1.72 km^2^ and the median is 0.18 km^2^. The location and child population aged under 15 years for each residential block were drawn from the 2010 Population Census (latest data as at January 17, 2017, S2 Table) [8].

The latitude and longitude of 803 hospitals and 217,186 residential blocks were determined using ArcGIS version 10.4 software (Esri, Redlands, CA, USA). Residential blocks from which hospitals were reachable by automobile within 30 and 60 minutes were calculated with Dijkstra’s algorithm using ArcGIS Online (Esri, Redlands, CA, USA) from February 5–22, 2017 [9]. Software presets for road data and the average velocity of an automobile (according to the type and width of roads in urban and rural areas) were used to calculate transportation time to hospitals (Table 2) [6]. ArcGIS Online (http://www.arcgis.com/index.html) offers a fee-based service (Service area analysis) that identifies areas that can reach a facility within a certain time. This service does not provide raw data of road networks; however, the latest road information used in this service is available through purchase from ESRI Japan as "ArcGIS Geo Suite Road Network" (https://www.esrij.com/products/data-content-geosuite-douromo/).

**Table 2:** Mean velocity of an automobile according to the type and width of roads (km/h).

		Width (m)				
	Type	≥13	5.5–13	3–5.5	<3	Unknown
Urban	Highway					
	(Inter-city)	80	80	50	10	2
	(Intra-city)	60	60	50	10	2
	Main road					
	(National)	30	20	17	7	2
	(Prefectural and municipal)	30	17	17	7	2
	Other road and unknown	30	12	8	4	2
Rural 1	Highway					
	(Inter-city)	80	80	60	15	10
	(Intra-city)	60	60	60	15	10
	Main road					
	(National)	50	40	25	10	10
	(Prefectural and municipal)	50	35	25	10	10
	Other road and unknown	50	20	15	10	10
Rural 2	Highway					
	(Inter-city)	80	80	60	15	10
	(Intra-city)	60	60	60	15	10
	Main road					
	(National)	55	50	30	10	10
	(Prefectural and municipal)	55	45	30	10	10
	Other road and unknown	55	30	15	10	10

Road density: Urban, ≥15,000 m/km^2^; Rural 1, 5,000~15,000 m/km^2^; Rural 2, <5,000 m/km^2^.

**Fig 1:**
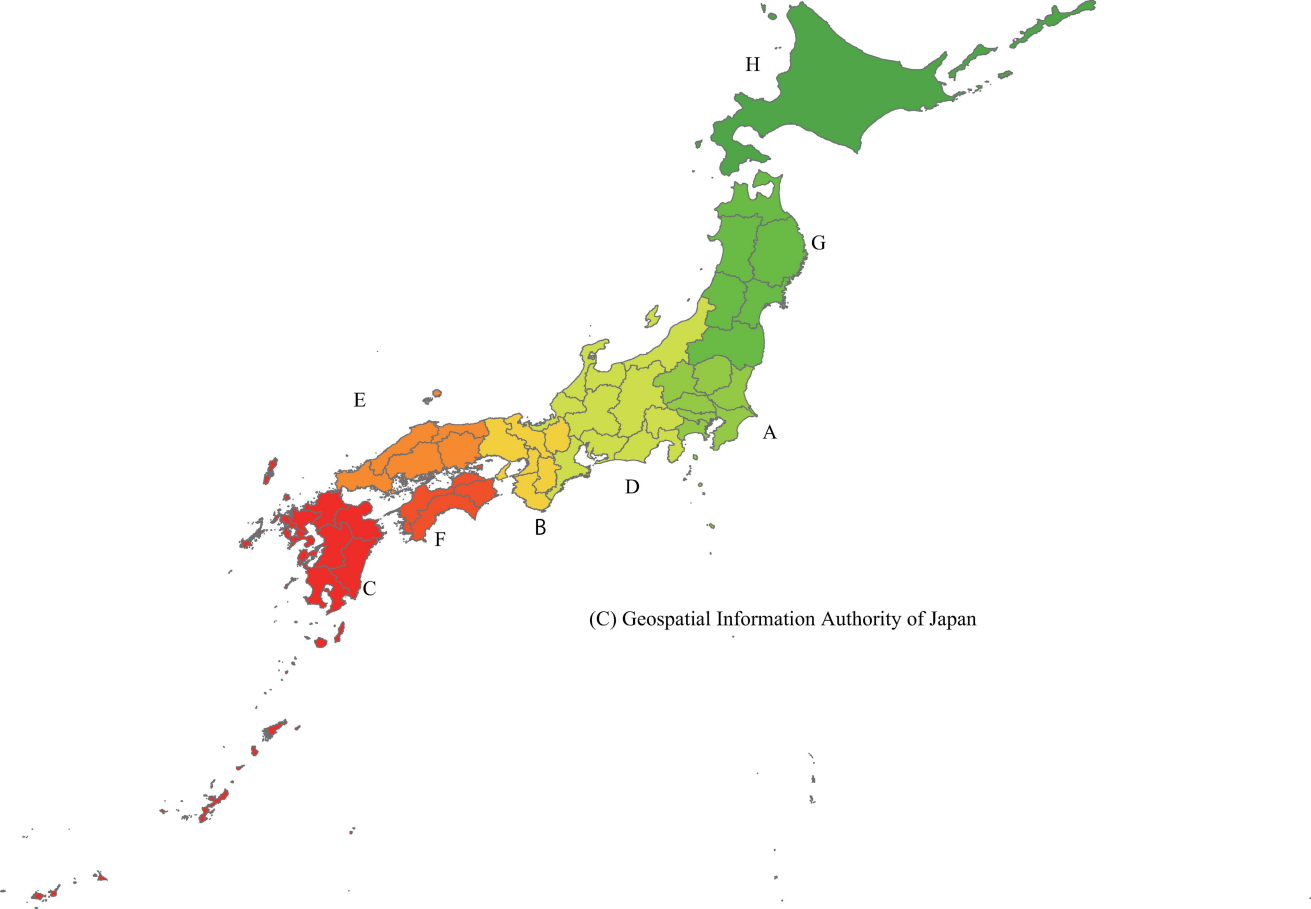
Japan’s eight regions. A, Kanto; B, Kinki; C, Kyushu and Okinawa; D, Chubu; E, Chugoku; F, Shikoku G, Tohoku; H, Hokkaido. Kanto and Kinki have high population density. Hokkaido, Tohoku, Shikoku, Chugoku, Chubu, and Kyushu and Okinawa have low population density. Map of Japan reprinted from Global Map Japan (public domain, open-access resources) under a CC BY license, with permission from the Geospatial Information Authority of Japan.

Japan is divided into eight regions (Fig 1, Table 3). In 2014, Japan’s population density was 341 persons/km^2^ (Table 3) [10]. Kanto (including Tokyo and Yokohama: 1,320 persons/km^2^) and Kinki (including Osaka, Kyoto, and Kobe: 759 persons/km^2^) have high population density, whereas the other six regions have low density (Hokkaido, 69 persons/km^2^; Tohoku, 135 persons/km^2^; Shikoku, 206 persons/km^2^; Chugoku, 233 persons/km^2^; Chubu, 321 persons/km^2^; Kyushu and Okinawa, 325 persons/km^2^). Hokkaido accounts for 21.0% of Japan’s total area and Tohoku for 17.9%, but only 4.2% of the population lives in Hokkaido and 7.1% live in Tohoku. I compared the proportions of children younger than 15 years resident in areas accessible to hospitals within 30 and 60 minutes among these eight regions.

**Table 3:** Total population and land area of eight Japanese regions.

	Region	Population density	Total population		Total land area	
		(persons/km^2^)	(persons)	(%)	(km^2^)	(%)
	Year	2014	2014		2014	
A	Kanto	1,320	42,797,000	33.7	32,429	8.7
B	Kinki	759	20,750,000	16.3	27,351	7.3
C	Kyushu and Okinawa	325	14,480,000	11.4	44,514	11.9
D	Chubu	321	23,305,000	18.3	72,582	19.5
E	Chugoku	233	7,436,000	5.9	31,921	8.6
F	Shikoku	206	3,878,000	3.1	18,804	5.0
G	Tohoku	135	9,036,000	7.1	66,947	17.9
H	Hokkaido	69	5,400,000	4.2	78,421	21.0
	Total	341	127,082,000	100.0	372,969	100.0

Total land area excluding the northern territories and Takeshima Island.

This study only used previously published data obtained from the Japanese government; therefore, ethical approval from the Medical Research Ethics Committee of Hiroshima International University was not required.

## Results

**Fig 2:**
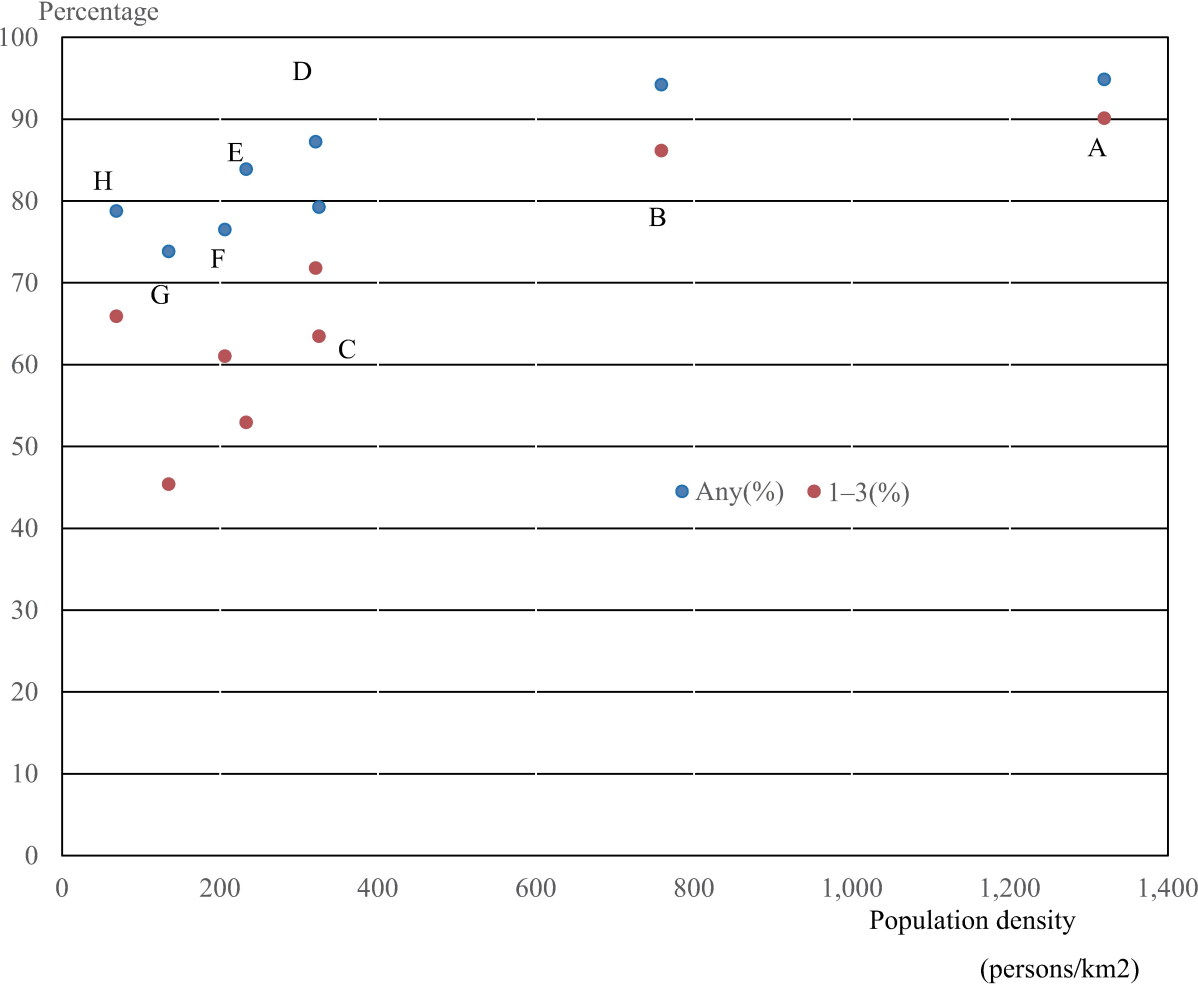
Population density and percentage of child population in areas accessible to hospitals meeting criteria 1–3 (red) and any one (blue) within 30 minutes by automobile. A, Kanto; B, Kinki; C, Kyushu and Okinawa; D, Chubu; E, Chugoku; F, Shikoku G, Tohoku; H, Hokkaido.

**Fig 3:**
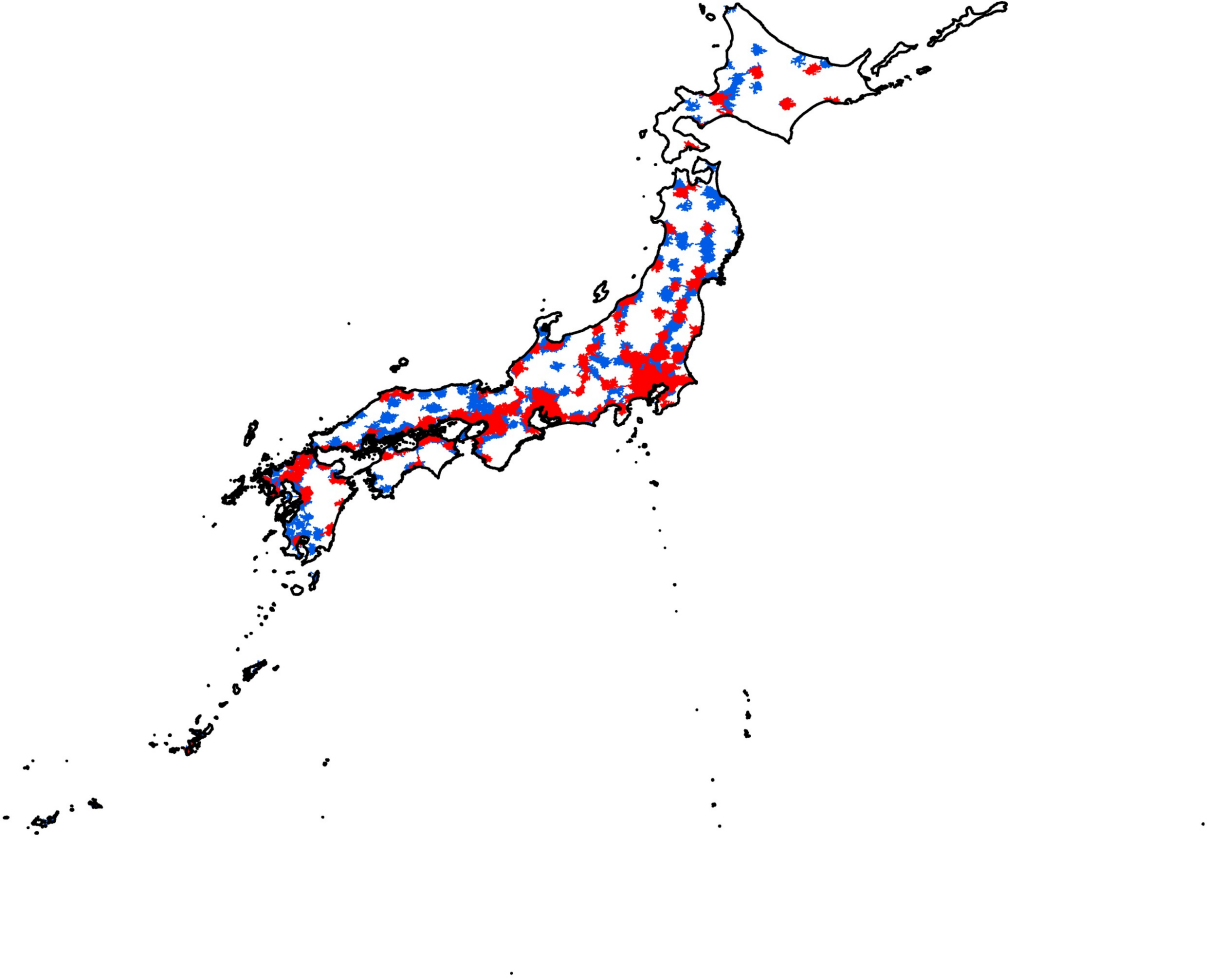
Areas accessible to pediatric inpatient service providers meeting criteria 1–3 (red) and 4–5 (blue) within 30 minutes by automobile.

In total, 88.0% of children in Japan lived in areas accessible to hospitals that met one of the high reimbursement criteria for pediatric inpatient services within 30 minutes. These areas represented 25.7% of Japan’s total land area (S3 Table). The proportion of children who could reach hospital within 30 minutes was higher in regions with high population density (e.g., Kanto and Kinki) compared with regions with low population density (Table 4, Figs 2 and 3). In addition, 75.5% of the child population lived in areas that could reach facilities in which five or more full-time pediatricians worked (criteria 1–3) within 30 minutes (equivalent to 13.3% of the total land area, S3 Table). The proportion of children with access to these hospitals was also higher in regions with high population density (Table 4, Figs 2 and 3).

**Table 4:** Percentage of land area and child population in areas accessible to hospital by automobile within 30 minutes.

		Child population		Land areas	
		Criteria for higher reimbursement			
	Region	Any (%)	1–3 (%)	Any (%)	1–3 (%)
A	Kanto	94.9	90.1	51.8	39.7
B	Kinki	94.2	86.2	41.7	22.2
C	Kyushu and Okinawa	79.2	63.5	28.7	13.2
D	Chubu	87.2	71.8	31.7	17.4
E	Chugoku	83.9	53.0	31.5	12.7
F	Shikoku	76.5	61.0	22.7	13.3
G	Tohoku	73.8	45.4	18.9	5.9
H	Hokkaido	78.8	65.9	7.3	2.8
	Total	88.0	75.5	25.7	13.3

**Fig 4:**
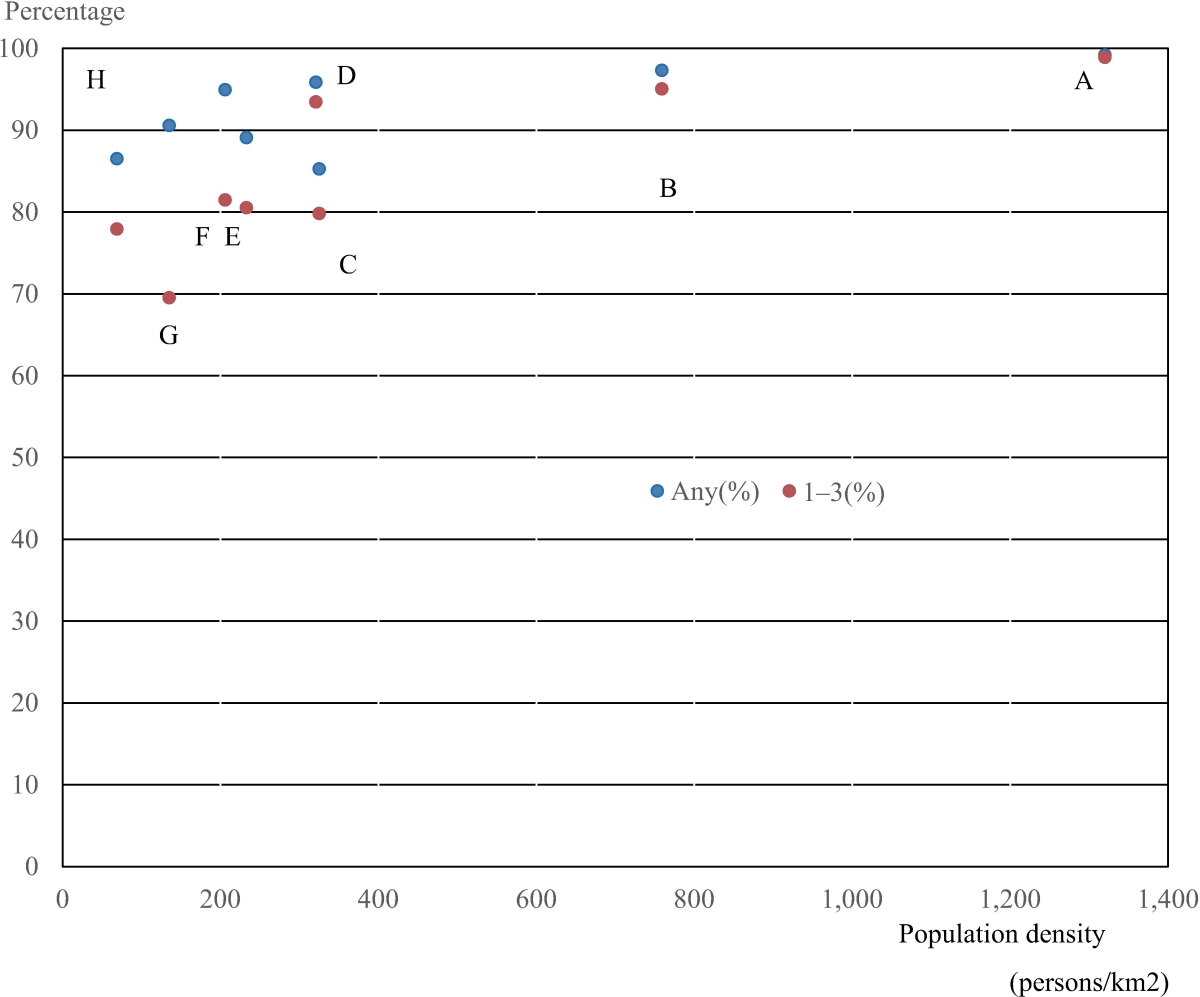
Population density and percentage of child population in areas accessible to hospitals meeting criteria 1–3 (red) and any one (blue) within 60 minutes by automobile. A, Kanto; B, Kinki; C, Kyushu and Okinawa; D, Chubu; E, Chugoku; F, Shikoku G, Tohoku; H, Hokkaido.

**Fig 5:**
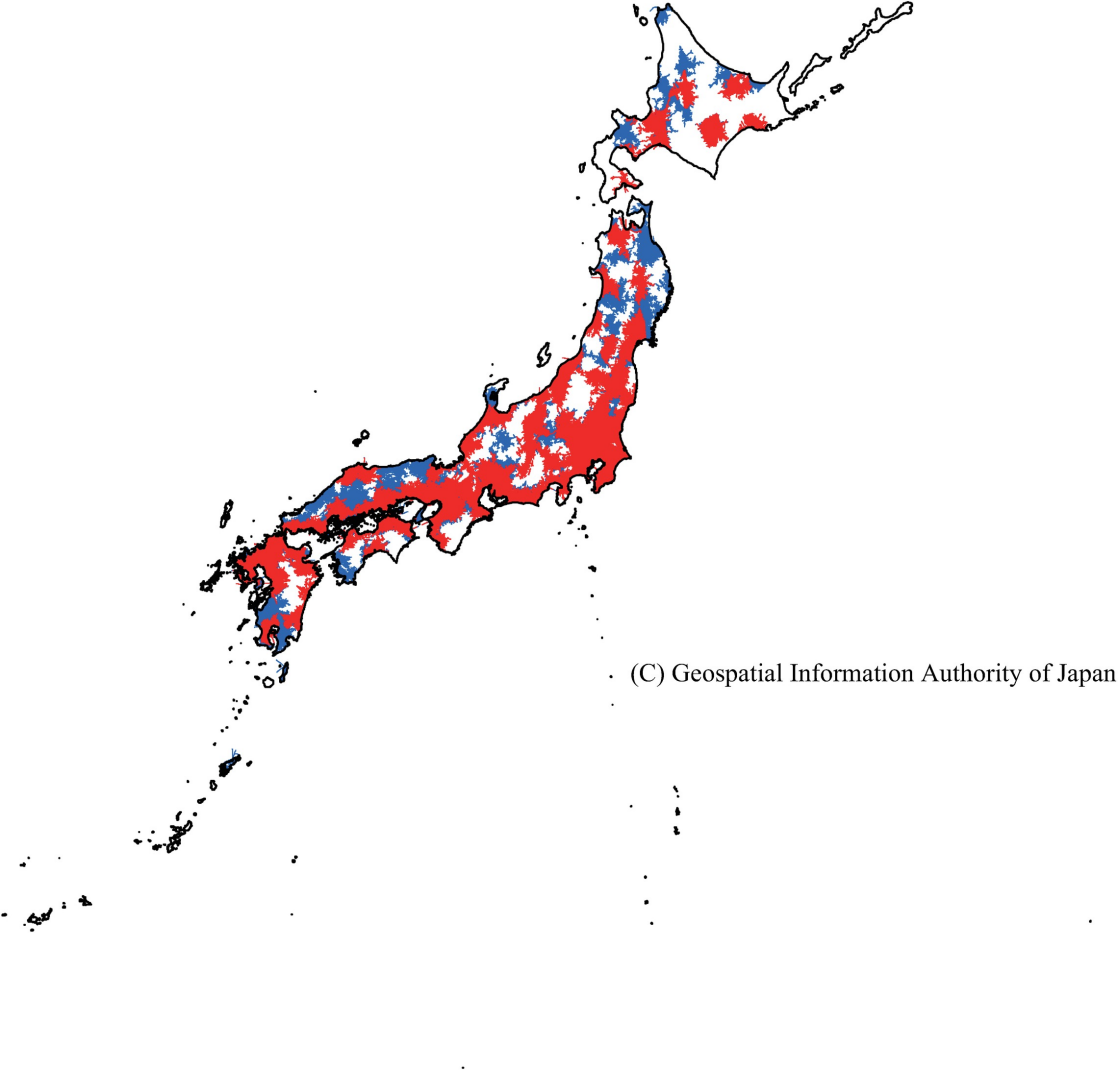
Areas accessible by automobile to pediatric inpatient service providers meeting criteria 1–3 (red) and 4–5 (blue) within 60 minutes.

In addition, 95.2% of children lived in areas accessible to hospitals that met one of the high reimbursement criteria within 60 minutes; 90.5% could access hospitals with pediatric departments that met criteria 1–3 within this time (S4 Table, Table 5, Figs 4 and 5). The percentage of children resident in areas accessible to hospitals that met one of the criteria within 60 minutes was high in regions with high population density, such as Kanto (99.2%) and Kinki (97.3%), and was relatively high in other regions (from 85.3% in Shikoku to 95.9% in Chubu). However, lower proportions of children lived in areas accessible to facilities that met criteria 1–3 within 60 minutes in regions with low population density (e.g., Hokkaido, 77.9%; Tohoku; 69.5%; Shikoku, 79.8%) compared with other regions (Table 5, Figs 4 and 5).

**Table 5:** Percentage of land area and child population in areas accessible to hospital by automobile within 60 minutes.

		Child population		Land areas	
		Criteria for higher reimbursement			
	Region	Any (%)	1–3 (%)	Any (%)	1–3 (%)
A	Kanto	99.2	98.9	85.2	77.3
B	Kinki	97.3	95.1	74.8	54.3
C	Kyushu and Okinawa	89.1	80.5	63.6	40.9
D	Chubu	95.9	93.5	69.5	53.4
E	Chugoku	95.0	81.5	79.1	40.6
F	Shikoku	85.3	79.8	51.8	39.3
G	Tohoku	90.6	69.5	53.9	24.2
H	Hokkaido	86.5	77.9	28.0	15.3
	Total	95.2	90.5	58.6	38.7

## Discussion

Previously, I calculated the linear distance from each residential block to the nearest hospital that met high reimbursement criteria for secondary and tertiary pediatric inpatient services [5]. Hospitals with at least one full-time pediatrician are able to receive medical fees based on one of five high reimbursement criteria. In Japan, children are able to visit secondary and tertiary medical centers without a referral letter, and there is no clear distinction between secondary and tertiary facilities. However, as provision of tertiary care requires more pediatricians, hospitals that meet criteria 1–3 may mainly provide tertiary services, whereas those meeting 4–5 may mainly provide secondary services. Predicting the time required to transport sick children to these facilities required data for road mileage and velocity of automobiles.

Japanese citizens have free access to medical facilities, which allow patients to choose any clinic or hospital. At night and during holidays, however, most patients are not able to visit their preferred clinic or hospital because these facilities do not always provide medical services. Therefore, in this study, I calculated the time required to visit the nearest hospital providing pediatric inpatient services from each residential block, using data for the average velocity of an automobile (according to the type and width of roads in urban and rural areas). This showed that 88.0% of children in Japan are able to access hospitals that meet one of the high reimbursement criteria for pediatric inpatient services within 30 minutes, and 95.2% within 60 minutes. Options for transporting children who need inpatient care in major cities and rural areas in Japan include private cars, taxis, and ambulances; it is unlikely that other forms of transport such as walking, bicycles, or trains are used in such circumstances. According to a recent Patient Survey, 92,000 children aged under 15 years were discharged from Japanese hospitals in September 2014 [11]. In addition, 471,000 children were transported by ambulance between January 1, 2014 and December 31, 2014 [12]. This suggests many children in Japan who need inpatient treatment access hospital by automobile (including ambulances), meaning this analysis based on automobile velocity is appropriate. Most children lived in areas from which pediatric inpatient services were accessible within 60 minutes. Although the number of hospitals with pediatric departments has decreased (from 4,120 in 1990 to 2,656 in 2014) [4], hospital accessibility for children within 60 minutes is likely to be maintained. This secure access is not limited to pediatrics, as 97.8% of women in Japan can access their nearest regional perinatal center within 60 minutes [13].

The proportion of children who could access hospitals meeting one of the high reimbursement criteria within 60 minutes did not differ markedly among the eight regions. However, the proportion of children who could access hospitals meeting criteria 1–3 (five or more full-time pediatricians) and estimated to provide tertiary services was lower in regions with low population density compared with regions with high population density. About 80% of the total person-days for pediatric inpatient care were estimated to be in hospitals that received medical fees based on high reimbursement criteria [14]. This indicates that this study appropriately considered children’s access to the nearest hospital that provided secondary and tertiary pediatric inpatient care.

In Japan, a worker’s death due to stroke or ischemic heart disease can be considered an industrial accident from overwork if they worked more than 252 hours/month [15]. Concentration of medical resources into regional pediatrics centers to help decrease pediatricians’ work hours is essential to prevent similar issues related to overwork.

Most sick children in Japan are treated by pediatricians rather than by general physicians. In 2014, 29,878 physicians had a main or minor specialty in pediatrics, whereas only 179 physicians were general practitioners and family physicians [16]. In addition, most hospitals with pediatric departments provide pediatric emergency services. Hospitals with few pediatricians are unlikely to be able to sustain 24-hour, 365-day secondary and tertiary pediatric medical services without the help of physicians with other specialties. In 2014, Japan had only 2.35 pediatricians per 10,000 population [16], meaning employing sufficient pediatricians to provide 24-hour, 365-day services in each municipality may be challenging, especially in under-populated areas [17]. Therefore, establishing secondary or tertiary pediatric centers in each municipality may not be practical. It is necessary to transport children with severe conditions to tertiary centers rather than treating them in small-scale pediatric departments, as the fatality rate of children treated by pediatric critical medicine specialists is lower than that for other specialties [18]. It is also essential to concentrate human resources (including pediatricians and nurses) in pediatric centers operated for children in areas that cover several cities, towns, and villages. However, it is important to construct appropriate transport systems.

Providing 24-hour, 365-day pediatric services may become a political promise, but provision of such services requires employment of a large number of pediatricians and nurses. Politicians should refrain from requiring hospital administrators to provide such services unless provision is made to employ sufficient medical staff, including pediatricians and nurses. Despite the decrease in number of hospitals with pediatric departments, this study demonstrated that most children can access medical facilities that provide secondary or tertiary pediatric services within 60 minutes by automobile.

## Limitations

1. Japanese citizens have free access to medical facilities, which allows patients to choose any clinic or hospital. However, the accessibility of medical facilities other than the nearest hospital was not analyzed.
2. As only data for the average velocity of an automobile were used, effects of factors such as traffic jams and climate worsening were not considered. However, the percentage of children resident in areas from which secondary or tertiary pediatric inpatient services could be accessed within 30/60 minutes did not differ markedly across regions. Therefore, it is likely that most children could reach such hospitals within 60 minutes even if the transportation velocity was reduced.
3. Each high reimbursement criterion for secondary and tertiary pediatric inpatient services only shows the minimum number of full-time pediatricians; the exact number is unknown.
4. I calculated transportation time to the nearest hospital from each residential block. Transportation time from other places was unknown. However, children’s homes are likely to be located near most facilities (e.g., nursery schools, kindergartens, and schools). Therefore, the approximation of transport time in this study may not cause serious issues.

## Conclusions

To provide effective pediatric services, the Japanese government promoted concentration of medical resources into “regional pediatrics centers,” which provide secondary or tertiary pediatric inpatient services and primary care at night and during holidays. Around 95.2% of children in Japan can reach such facilities within 60 minutes by automobile, although this proportion is lower in regions with low population density.

## Supporting information

S1 Table Latitude and longitude of 803 hospitals that met one of the high reimbursement criteria for pediatric inpatient services. (XLSX)

S2 Table Location and child population aged under 15 years for each residential block. (XLSX)

S3 Table Residential blocks accessible to a hospital meeting criteria 1–3 and any one by automobile within 30 minutes. (XLSX)

S4 Table Residential blocks accessible to a hospital meeting criteria 1–3 and any one by automobile within 60 minutes. (XLSX)
